# Effect of Increasing the Strength of Aluminum Matrix Nanocomposites Reinforced with Microadditions of Multiwalled Carbon Nanotubes Coated with TiC Nanoparticles

**DOI:** 10.3390/nano9111596

**Published:** 2019-11-11

**Authors:** Artemiy Aborkin, Kirill Khorkov, Evgeny Prusov, Anatoly Ob’edkov, Kirill Kremlev, Igor Perezhogin, Michail Alymov

**Affiliations:** 1 Vladimir State University Named after Alexander and Nikolay Stoletovs, Gorky Str. 87, Vladimir 600000, Russia; freeod@mail.ru (K.K.); eprusov@mail.ru (E.P.); 2 G. A. Razuvaev Institute of Organometallic Chemistry of the Russian Academy of Sciences, Tropinina Str. 49, Nizhny Novgorod 603950, Russia; amo@iomc.ras.ru (A.O.); kkremlev@mail.ru (K.K.); 3 Technological Institute for Superhard and Novel Carbon Materials, Tsentral’naya Str. 7a, Troitsk 142190, Russia; iap1@mail.ru; 4 Merzhanov Institute of Structural Macrokinetics and Materials Science of the Russian Academy of Sciences, Academician Osipyan Str. 8, Chernogolovka 142432, Russia; isman@ism.ac.ru

**Keywords:** aluminum matrix nanocomposites, MWCNTs, titanium carbides, powder metallurgy, mechanical properties, interface, microstructure

## Abstract

Aluminum matrix composites reinforced with multiwalled carbon nanotubes (MWCNTs) are promising materials for applications in various high-tech industries. Control over the processes of interfacial interaction in Al/MWCNT composites is important to achieve a high level of mechanical properties. The present study describes the effects of coating MWCNTs with titanium carbide nanoparticles on the formation of mechanical properties and the evolution of the reinforcement structure in bulk aluminum matrix nanocomposites with low concentrations of MWCNTs under conditions of solid-phase consolidation of ball-milled powder mixtures. Using high-energy ball milling and uniaxial hot pressing, two types of bulk nanocomposites based on aluminum alloy AA5049 that were reinforced with microadditions of MWCNTs and MWCNTs coated with TiC nanoparticles were successfully produced. The microstructural and mechanical properties of the Al/MWCNT composites were investigated. The results showed that, on the one hand, the TiC nanoparticles on the surface of the MWCNT hybrid reinforcement reduced the damage of reinforcement under the intense exposure of milling bodies, and on the other hand, they reduced the contact area of the MWCNTs with the matrix material (acting as a barrier interface), which also locally inhibited the reaction between the matrix and the MWCNTs.

## 1. Introduction

Aluminum matrix composites reinforced with carbon nanotubes (CNTs) have significant potential for applications in automotive, aerospace, and other high-tech industries: this is driven by the possibility of achieving high physical and mechanical properties of products even at a low content of the reinforcing phase due to the extended specific surface of the reinforcement and the effects associated with nanostructuring [1,2,3]. The intensification of research and development in the field of Al/CNT composites has largely been facilitated by growth in the world’s industrial production of carbon nanotubes [4] as well as by successes achieved in the direction of their practical use in various industries [5]. To date, a wide range of technological methods have been tested to obtain Al/CNT composites, such as various methods of powder metallurgy, deformation and thermal deformation processing, and electrochemical and laser deposition [6].

The positive effect of carbon nanotubes on the mechanical properties (tensile strength, compressive strength, flexural strength, etc.) of aluminum matrix nanocomposites has been described in many works [7,8,9]. However, the actually achieved indicators of the strength characteristics of Al/CNT composites remain below those theoretically predicted, which is due to the uneven distribution of reinforcement in the matrix, the unsatisfactory state of the interphase boundaries, and other factors. One of the main problems that accompanies the preparation of aluminum matrix composites with carbon nanotubes and limits their practical application is the difficulty of achieving strong interfacial bonding between nanotubes and an aluminum matrix. This is associated with poor wettability of the surface of solid carbon by metal melts in liquid-phase technologies [10] as well as with the presence of a natural oxide layer on the surface of aluminum particles that impedes direct contact with nanotubes in solid-phase technologies of Al/CNT composite manufacturing [11]. At the same time, a strong and stable adhesive bond is a significant factor for increasing the mechanical properties of composites up to their theoretical limit [12]. Therefore, the control of interfacial reactions (allowing for a certain interfacial interaction, but only to a limited extent) will improve interfacial bonding and correspondingly increase effective strength, as shown in Reference [13].

Undesirable products at interfaces leading to degradation of the reinforcement are another important problem in the technology of producing composites with carbon nanotubes [14]. The following approaches have been used to solve the indicated problem: the surface modification of nanotubes by chemical oxidation [15], direct in situ synthesis of carbon nanotubes on aluminum powder particles [16], and controlled formation of an aluminum carbide layer at the Al/CNT interface [17]. Hybridization of the reinforcement through the formation of nanosized particles or continuous coatings on the CNT surfaces, which appear as an interphase barrier layer at the Al/CNT interface, can significantly increase adhesion with the matrix and contribute to a more efficient load redistribution, which ensures a higher level of mechanical properties [18].

To inhibit the processes of degradation of the reinforcement and increase the adhesive bond at the interfaces, carbon nanotubes are decorated or coated with metal, carbide, or oxide phases [19,20,21,22]. As practice has shown, many of the above technological problems can be successfully solved by decorating the CNT surfaces with titanium carbide nanoparticles. The layer of TiC particles prevents the structural degradation of CNTs in contact with the matrix and other media, acting as a diffusion barrier [23]. Despite the significant prospects for such an approach, data on the synthesis and properties of aluminum matrix composites with carbon nanotubes decorated with titanium carbide have been limited in the literature so far. Among the published papers, the works of F. Saba et al. [24,25,26] should be noted. However, in these works, unalloyed aluminum was used as the matrix material. When speaking about practical applications, aluminum-based alloys are of more interest. In particular, AA5049 can be used in the automotive industry [27].

As is known, the introduction of a large fraction of the reinforcement along with increased strength leads to embrittlement of the composite material. An increase in strength with a slight decrease in ductility is possible through the addition of carbon nanotubes in small concentrations. Besides, the presence of an additional operation to modify nanotubes significantly increases their cost, which leads to an increase in the cost of the final product from aluminum matrix nanocomposites. This makes studying the effect of microadditives of carbon nanotubes on the properties of aluminum matrix composites justified from a scientific, technical, and economic point of view. Microadditions of carbon nanotubes contribute to an increase in strength characteristics of the material, and at the same time they maintain sufficient ductility, reduce the agglomeration of the reinforcement, and provide a greater economic effect.

The purpose of this work was to study the effect of coating multiwalled carbon nanotubes with titanium carbide nanoparticles on the formation of mechanical properties and the evolution of the reinforcement structure in bulk aluminum matrix nanocomposites with low concentrations of carbon nanotubes under conditions of solid-phase consolidation of ball-milled powder mixtures.

## 2. Materials and Methods

### 2.1. Materials Processing

The initial matrix material included granules of aluminum alloy AA5049 (1–2 mm in size). The elemental composition of the granules was measured using an ARL ADVANT’X (Thermo Fisher Scientific Inc., Waltham, MA, USA) sequential X-ray fluorescence spectrometer as follows (wt %): Al 96.11; Mg 2.42; Mn 0.55; Fe 0.26; Si 0.37; Zn up to 0.1; Ti up to 0.1; and Cu to 0.1.

As reinforcing additives, two types of carbon nanostructures were used for comparison: multiwalled carbon nanotubes (MWCNTs) and multiwalled carbon nanotubes coated with titanium carbide nanoparticles (TiC/MWCNTs).

The initial MWCNTs were synthesized through the metal-organic chemical vapor deposition (MOCVD) method with ferrocene and toluene used as precursors under an argon flow in a tubular reactor equipped with a tubular furnace at 825 °C, as described in detail elsewhere [28]. The argon flow rate was 450 cm^3^/min. The time for MWCNT synthesis was 5 h, and the average diameter of the carbon nanotubes obtained under these conditions was on the order of 70 nm. For the obtained nanotubes, a detailed characterization was carried out using SEM and HRTEM methods.

A titanium carbide coating on the surface of the nanotubes was formed as follows: a preset amount of MWCNTs (not exceeding 400 mg) was placed on a special stainless steel grid at the center of a silica reactor, and a corresponding amount (within 1–3 g, depending on the desired TiC layer thickness) of bis(cyclopentadienyl)titanium dichloride (metalorganic precursor compound) was placed on the reactor bottom. The reactor was preliminarily pumped to the fore-vacuum level, after which the central zone (where MWCNTs occurred) was gradually heated to 900 °C. Then, the precursor evaporator was heated to 160 °C, and the vapor of the precursor was admitted to the zone of pyrolysis. The pyrolysis of the precursor vapor took place on the MWCNT surfaces with the formation of a TiC/MWCNT hybrid nanomaterial representing carbon nanotubes with a surface modified by a TiC coating. The other products of pyrolysis were continuously removed from the reaction zone by a vacuum pump and frozen in a liquid nitrogen-cooled trap [29].

**Figure 1:**
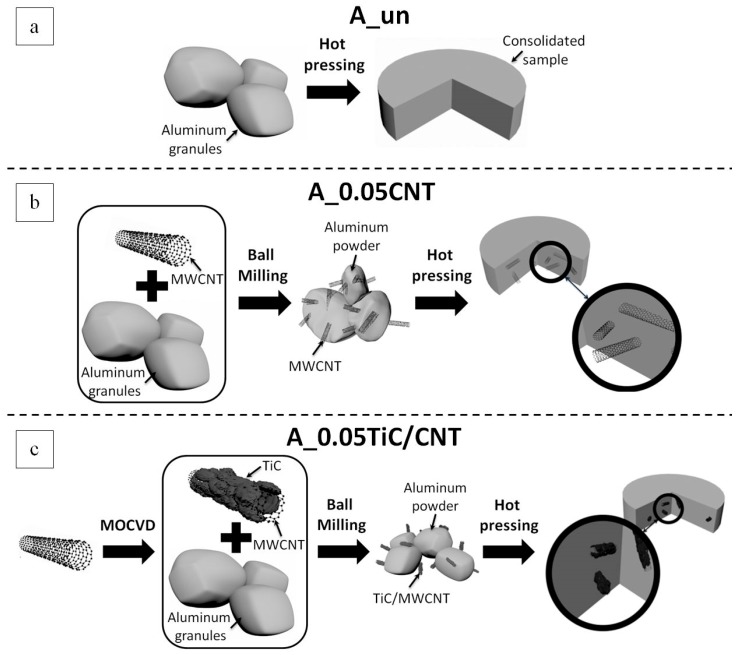
Schematic representation of the synthesis of bulk materials: (**a**) producing consolidated samples (A_un) from the initial aluminum granules without ball milling; (**b**) producing consolidated samples of aluminum matrix nanocomposites (A_0.05CNT) reinforced with carbon nanotubes without prior coating; (**c**) producing consolidated samples of aluminum matrix nanocomposites (A_0.05TiC/CNT) reinforced with carbon nanotubes and coated with titanium carbide nanoparticles.

A schematic representation of the process of producing bulk materials for comparative studies is shown in Figure 1.

AA5049 aluminum alloy granules were mixed with reinforcing additives. The mass fraction of the reinforcement, the process control agents, and abbreviations for the studied compositions of materials are shown in Table 1.

The mechanical processing for the synthesis of powder mixtures was carried out in a 1000-ml stainless steel water-cooled vial at 600 rpm using an Activator 4M planetary ball mill (Chemical Engineering Plant LLC, Dorogino, Russian Federation). The processing time was 6 h. Stearic acid was used as a process control agent. Balls of hardened steel (100Cr6) with a diameter of 10 mm were used as milling bodies with the ratio of balls/charge at 5:1.

The milled powders were cold-compacted in a hydraulic uniaxial press at 350 MPa to obtain briquettes with a diameter of 41 mm. The resulting briquettes were hot-pressed on the same press in a special heated steel mold at 450 °C and 400 MPa to obtain compacts with a diameter of 42 mm and a height of 20 mm.

### 2.2. Characterization Techniques

The powder morphology was characterized using a scanning electron microscope (the Quanta 200 3D). The samples were characterized in a transmission electron microscope (TEM) (JEM-2010, Jeol Ltd., Tokyo, Japan) equipped with an attachment for energy-dispersive X-ray spectroscopy (EDS). Low magnification images, high-resolution images and diffraction patterns, as well as EDS of the samples were obtained. The carbon structures in the raw MWCNTs and TiC/MWCNTs were prepared for TEM observation by milling the initial material in hard alloy anvils and then dispersing them in an ethanol solution. The resulting mixture was deposited on special copper TEM grids. Bulk samples were prepared by ion beam polishing in an Ion Slicer EM 09100IS (Jeol Ltd., Tokyo, Japan).

The granulometric composition of the powders was determined by dynamic light scattering (DLS) analysis by a Microsizer 201C (VA Instalt Ltd., St.-Petersburg, Russian Federation).

The carbon structures in the raw MWCNT and TiC/MWCNT powders and the bulk samples were investigated by Raman spectroscopy with an Ntegra Spectra (NT-MDT LLC, Moscow, Russian Federation), at 473 nm with no more than 50 mW. The Raman spectra were measured at more than 10 different points on each sample. The obtained spectra were fitted, and the integrated intensity of the D- and G-band of carbon (*I_D_/I_G_*) was calculated. To probe the homogeneity of the powder composites at the micron scale after ball milling, a statistical analysis of the change in *I_D_/I_G_* was performed. The registration of Raman spectra was performed along a line with a length of 100 μm at a step of 0.78 μm on the polished sections of cold-pressed powder samples.

The surfaces of the bulk samples were examined by atomic force microscopy (AFM) using an Ntegra Spectra (NT-MDT LLC, Moscow, Russian Federation) technique. AFM studies were carried out in semicontact modes with the cantilevers of NSG10-A.

The crystallite structure was investigated using powder X-ray diffraction by a D8 ADVANCE (BRUKER, Billerica, MA, USA) with Cu Kα (λ = 1.5148 Å, 40 kV, and 40 mA) in the 2θ range (20°–85°) using a linear detector. The phases were identified using the ICDD PDF-2 database. The crystallite size was determined based on the XRD (111) Al peak. The instrumental broadening was determined with an α-Al_2_O_3_ standard sample. The crystallite size was calculated using the Scherrer equation:

(1) $$d=\frac{0.9\lambda }{B\;\cos\theta },$$

where *d* is the crystallite size; and *λ*, *θ*, and *B* represent the X-ray wavelength, the Bragg scattering angle, and the full width at half maximum (FWHM), respectively.

Elemental oxygen contents were measured using an МЕТАВАК-АК (Eksan Ltd., Izhevsk, Russian Federation) infrared cell. The density of the bulk samples was measured using the Archimedes method with subsequent calculation of the average value from five measurements. During measurements, distilled water was used as a liquid with a known density.

The microhardness and elastic modulus of the powders and of the bulks were measured through the method of kinematic indentation using a Micro-Combi tester (CSM Instruments, Peseux, Switzerland). The measurements were performed with a Vickers pyramid-shaped indenter with a load of 0.1 N for 10 s. At least 10 indentation measurements per sample were conducted. The calculation of the modulus of elasticity was performed using the Oliver–Pharr method according to Reference [30].

Cylindrical compression specimens with a diameter of 5 mm and a gauge length of 9 mm were machined from consolidated samples. The compression specimens were prepared perpendicularly to the pressing direction, and the compression tests were carried out at room temperature using a universal testing machine (Z400, Zwick GmbH, Ulm, Germany) with a speed of 5 mm·min^−1^.

## 3. Results and Discussion

**Figure 2:**
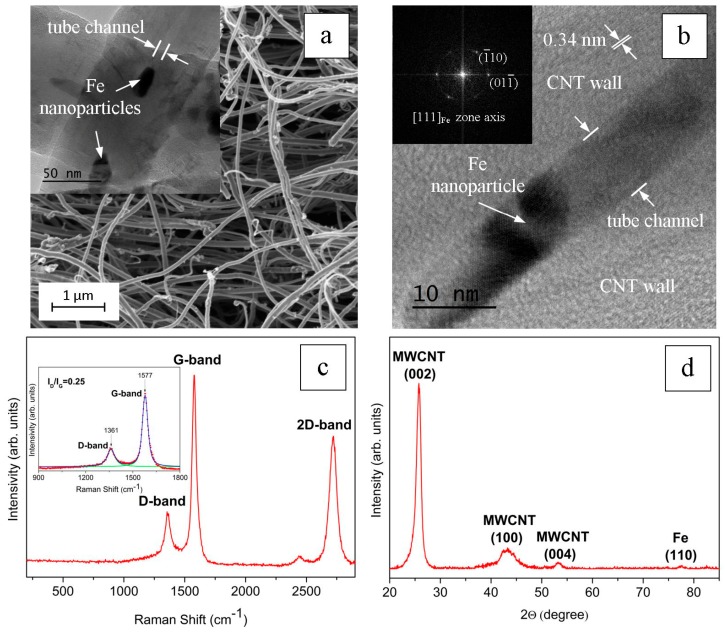
SEM image of MWCNTs (**a**). The inset in Figure 2a shows a TEM image of a nanotube with iron nanoparticles in the internal channel. An HRTEM image of an MWCNT with an iron nanoparticle in the inner channel (**b**): the Fourier transform of the image of the crystal lattice of iron is shown in the inset in the upper left corner in Figure 2b. In the inset in Figure 2a and in Figure 2b, the pointing arrows indicate iron nanoparticles, and the dimensional arrows indicate the width of the inner channel of the nanotube. Raman spectrum (**c**) and XRD pattern (**d**) of MWCNTs.

Figure 2a,b shows the results of the SEM and HRTEM studies of the initial MWCNTs. The average diameter of the MWCNTs was about 80 nm, and the length was from several hundred microns to several millimeters (see Figure 2a). The lateral surfaces of the MWCNTs were formed by graphene layers with distances between them of about 0.34 nm (see Figure 2b). The surfaces of the MWCNTs were not ideal: defects in the form of residual graphene layers were observed. In addition, the remains of iron catalyst nanoparticles were recorded in the internal channel of carbon nanotubes (see inset in Figure 2a). For example, Figure 2b shows an HRTEM image where an iron nanoparticle completely filled a part of the inner channel of the MWCNTs (with a diameter of about 10 nm). The zone axis of the body-centered (bcc) lattice of the iron nanocrystal was [111], and the contrast of the {110} atomic planes is visible in the image. The Fourier transform of the image of the iron crystal lattice is shown in the inset in the upper left corner of Figure 2b. The observed iron residues are a common byproduct of the MWCNT production technology.

A typical Raman spectrum of the initial MWCNTs is shown in Figure 2c. The band around D that can be observed around 1361 cm^−1^ corresponds to zone edge phonons and was activated through a mechanism of double resonance involving the elastic scattering of electrons through structural defects. The so-called 1577 cm^−1^ is called the G-band and corresponds to carbon–carbon stretching modes. Therefore, the intensity ratio *I_D_/I_G_* characterizes the defect density of the carbonaceous structures. The low *I_D_/I_G_* ratio (equal to 0.25) for these MWCNTs confirmed that their structure was not very defective, at least less so than the defectiveness of most industrially produced MWCNTs studied in the work of Reference [31].

The X-ray diffraction (XRD) analysis (Figure 2d) showed that the main MWCNT signatures corresponded to the graphite peaks (002), (004), and (100). On the other hand, diffraction peak (110) confirmed the presence of a residual iron catalyst.

**Figure 3:**
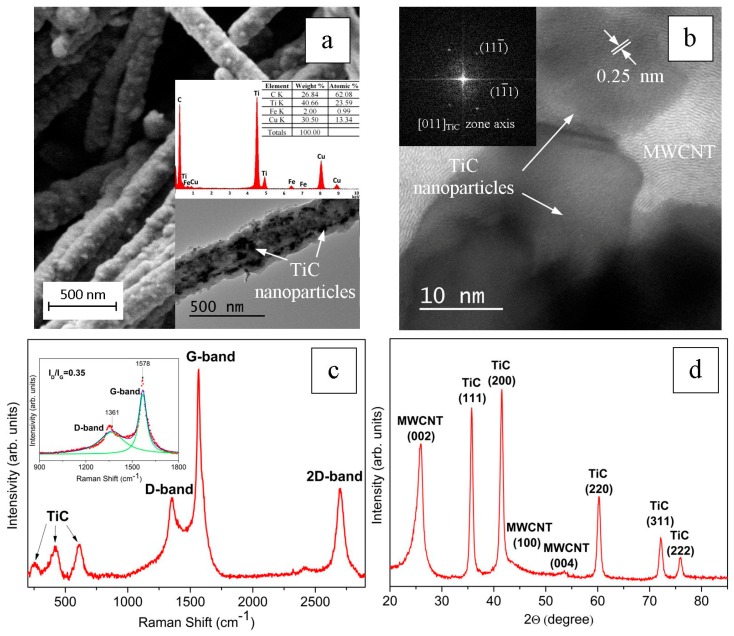
SEM image of MWCNTs coated with TiC nanoparticles (**a**): the inset in the lower right corner in Figure 3a shows a TEM image and EDS analysis data. An HRTEM image of an MWCNT with TiC nanoparticles in its walls (**b**): a Fourier image of the TiC crystal lattice image is shown in the inset in the upper left corner in Figure 3b. In the inset of Figure 3a and in Figure 3b, the pointing arrows indicate TiC nanoparticles. Raman spectrum (**c**) and XRD pattern (**d**) of an MWCNT coated with TiC nanoparticles.

Figure 3 shows the results of the characterization of MWCNTs coated with TiC nanoparticles. After MOCVD treatment of the initial MWCNTs, TiC nanoparticles with a characteristic size of 10–30 nm were observed on the surface. A TEM image of TiC/MWCNTs and EDS data is shown in Figure 3a. An HRTEM image, where the curved layers of graphite that make up the walls of the MWCNTs and the crystal lattice of titanium carbide particles that is embedded in these walls are visible, is shown in Figure 3b. The contrast of the planes {111} of this lattice is visible in the image. The corresponding Fourier transform is shown in the inset in the upper left corner of Figure 3b.

A typical Raman spectrum of MWCNTs coated with TiC nanoparticles is shown in Figure 3c. In addition to the D- and G-lines, the spectrum recorded lines corresponding to TiC (see Figure 3c). In this case, the *I_D_/I_G_* ratio increased compared to the initial MWCNTs up to 0.35, but nevertheless, it remained quite low.

The XRD analysis (Figure 3d) showed that the MWCNT hybrid material contained two phases—the graphite phase (as in the initial MWCNTs), and the coating substance—the cubic TiC phase.

**Figure 4:**
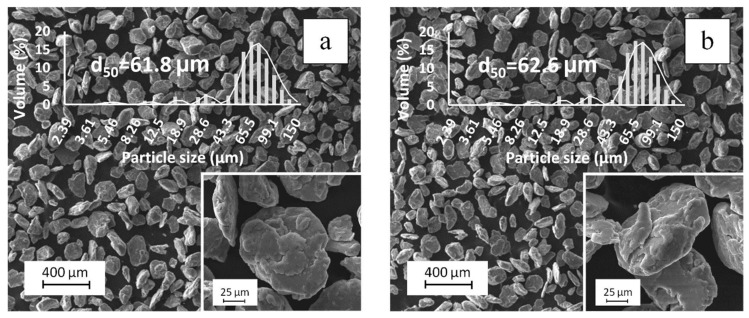
SEM images and dynamic light scattering (DLS) analysis of composite powders A_0.05CNT (**a**) and A_0.05TiC/CNT (**b**).

Figure 4 shows typical SEM images and the results of the DLS analysis of the A_0.05CNT and A_0.05TiC/CNT composite powders milled over 6 h with AA5049 matrix to obtain composite powders with 0.05 wt % additives. The morphology of all milled powders was equiaxial, and the mean particle size was in the range of 61.8–62.6 μm.

Reinforcement was observed in the form of separately lying MWCNTs with a length of 0.4 to 5 μm on the surface of A_0.05CNT powder particles. In A_0.05TiC/CNT powders, reinforcement was not recorded in SEM images. In our opinion, this does not indicate the destruction of TiC/MWCNTs under the high-energy impact of milling media, but is a feature of MWCNT hybrid structures, which may be associated with an improvement in the adhesive interaction of MWCNTs with matrix material due to the presence of a boundary layer in the form of TiC nanoparticles. As is known, the contact angle in the Al–TiC system [32] is ~2.5 times smaller than that in the Al–C system before the reaction of aluminum and carbon [33]. Therefore, taking into account that a developed plastic flow of aluminum occurs during high-energy ball milling, sticking of the matrix material to the surface of TiC/MWCNTs is possible even at the stage of processing in the mill, creating the bond “matrix material–reinforcing additive”. In this case, the nanotubes are “captured” inside the matrix material, which complicates their identification by SEM.

**Figure 5:**
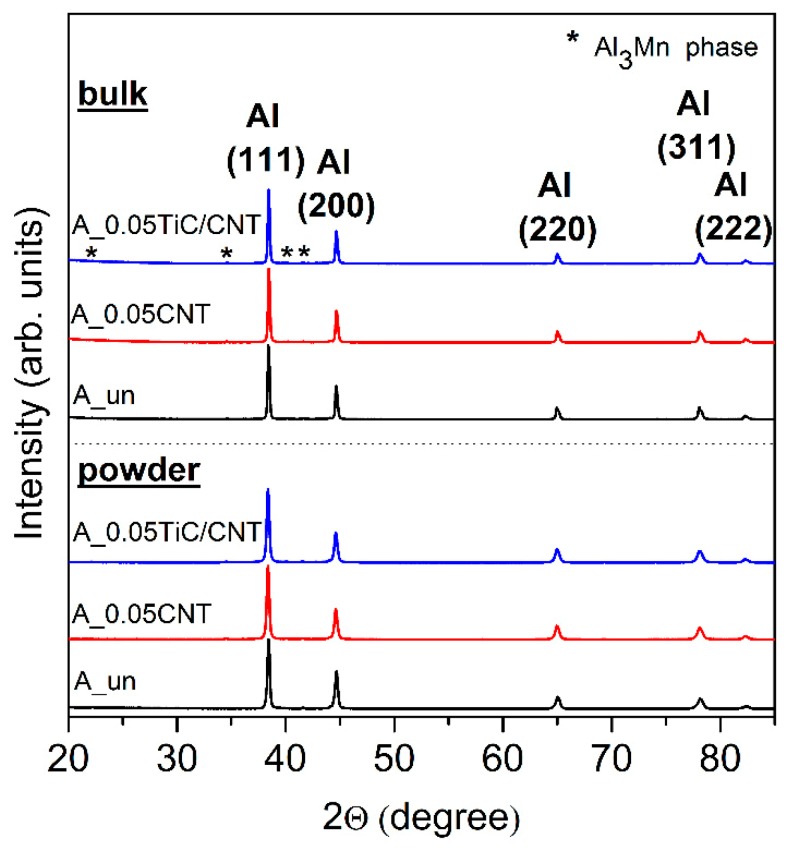
XRD patterns of powders and bulk materials: A_un, A_0.05CNT and A_0.05TiC/CNT.

The results of the X-ray diffraction analysis of the initial matrix material, milled powders, and samples obtained by hot pressing are presented in Figure 5. The diffraction patterns had a qualitatively similar character. Only diffraction peaks corresponding to the matrix material were observed, namely pronounced peaks related to the aluminum α-solid solution and low-intensity peaks, which were identified as the intermetallic phase Al_3_Mn. The diffraction peaks of MWCNTs and TiC/MWCNTs were not recorded, since the amount of reinforcing additives in the series of experiments was below the detection limit of XRD measurements.

It should be noted that the diffraction patterns did not have peaks corresponding to the formation of new phases, e.g., Al_4_C_3_ or Al_2_O_3_, both after ball milling and after hot pressing. However, XRD data alone cannot unambiguously confirm the absence of the formation of these phases. Taking into account the experimental results of many authors, for example [17,31,34,35,36,37], on the formation of Al_4_C_3_ and/or Al_2_O_3_ phases in composites reinforced with MWCNTs obtained by ball milling, the absence of these phases according to X-ray diffraction data, in our case, may have been associated with a limitation of the sensitivity of the XRD method in relation to the studied concentrations of additives.

The crystallite size calculation performed in accordance with equation (1) for powder composites showed that after ball milling, the crystallite size was 116.7 ± 3 nm and 125.5 ± 5 nm for the A_0.05CNT and A_0.05TiC/CNT powders, respectively. It should be noted that the achieved crystallite size was much higher than that reported in References [31,38,39]. This was due both to the type of matrix material and the conditions of ball milling used in this work, and it reflected the effect of the concentration of reinforcement on the decrease in grain size during processing in a ball mill. On the one hand, this should have reduced the mechanical properties of the composites, since the Hall–Petch reinforcing constituent would decrease. On the other hand, it had a positive effect on the ductility of the material.

After hot pressing, as expected, an increase in the FWHM of the Bragg diffraction peaks occurred. This indicated an increase in grain size during isothermal aging with hot pressing.

**Figure 6:**
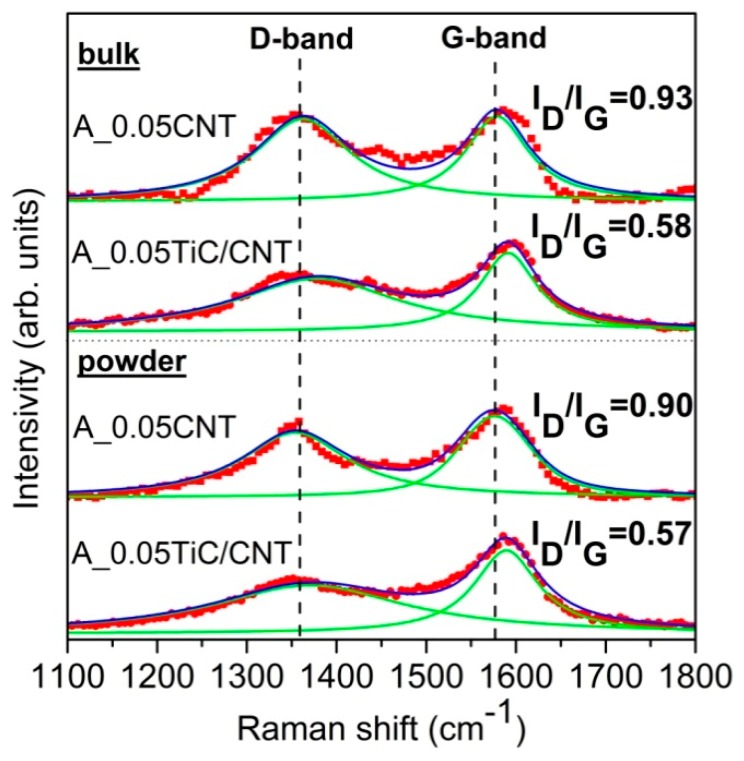
Raman analysis of powders and bulk materials: A_0.05CNT and A_0.05TiC/CNT.

The evolution of the structure of the reinforcement during milling with AA5049 was studied using Raman spectroscopy. Figure 6 shows typical Raman spectra obtained from composite powders after ball milling and on bulk composites. A comparison of the obtained spectra to the data shown in Figure 2c and Figure 3c indicated an increase in width and a decrease in the intensity of the G-band. Since the G-band is typically observed for perfect hexagonal graphite, the noted changes therefore indicated poor structuring (a violation of the structural order) and an increase in the defectiveness of the nanotubes during ball milling [40]. Despite a significant increase in the *I_D_/I_G_* ratio from 0.25 to 0.9 for A_0.05/CNT and from 0.35 to 0.57 for A_0.05TiC/CNT after ball milling and the preparation of the composite powders, the *I_D_/I_G_* ratio was low. Thus, for example, in Reference [38], the *I_D_/I_G_* ratio at a MWCNT concentration of 1 wt % reached a value of more than 1.2, and in Reference [31], the 1 vol % MWCNT *I_D_/I_G_* ratio was more than 2. Nevertheless, the authors of these studies showed (using TEM) that even for such high *I_D_/I_G_* ratios, MWCNTs were not completely destroyed and had a significant effect on increasing the strength of bulk composites. Thus, for the used nanotube concentrations, this convincingly confirmed that the nanotubes were preserved after mechanical milling and consolidation and allowed us to conclude that the presence of titanium carbide as a coating agent led to less damage to the nanotubes in the processes of obtaining powder and bulk nanocomposite materials.

For the bulk composite materials A_0.05CNT and A_0.05TiC/CNT, the *I_D_/I_G_* ratio slightly increased from 0.90 to 0.93 and from 0.57 to 0.58, respectively. On the one hand, the obtained values fit into the statistical error, and on the other hand, this (at least indirectly) could indicate additional damage of the reinforcement during hot pressing that was associated, e.g., with the in situ formation of Al_4_C_3_. These patterns were similar for both powder and bulk materials.

**Figure 7:**
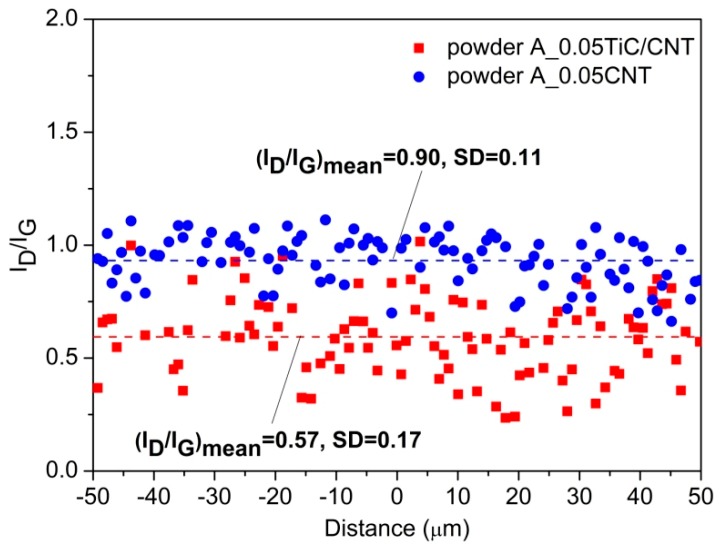
Typical *I_D_/I_G_* Raman intensity ratio measured over lines of longitudinal cross-sections: A_0.05CNT and A_0.05TiC/CNT composite powders.

In addition, it was interesting that, judging by the *I_D_/I_G_* ratio, the damage during ball milling of the initial MWCNTs was higher than for the TiC/MWCNTs (*I_D_/I_G_* = 0.9 versus *I_D_/I_G_* = 0.57). To confirm the noted regularity on a micron scale, linear micromapping was performed according to the technique used in References [41,42]. Figure 7 shows the distribution of the *I_D_/I_G_* ratio obtained for the composite powders at a scanning length of 100 μm. It can be seen from the presented data that the average *I_D_/I_G_* ratio was 0.90 at SD 0.11 and 0.57 at SD 0.17 for the A_0.05CNT and A_0.05TiC/CNT powders, respectively. This also indicates the positive effect of the MWCNTs coated with ceramic TiC nanoparticles on the integrity of the carbon nanostructures.

**Figure 8:**
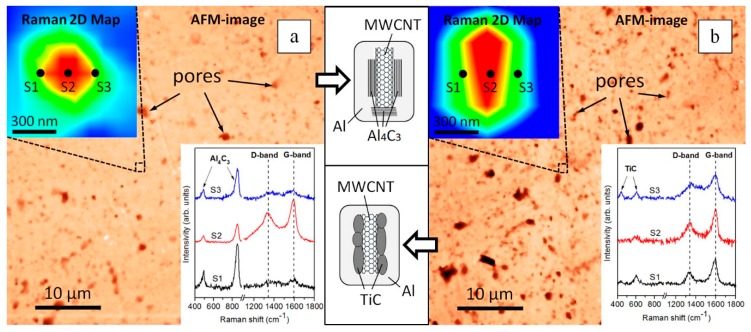
Atomic force microscopy (AFM) images of the surface, 2D mapping, and Raman spectra of the bulk composites: A_0.05CNT (**a**) and A_0.05TiC/CNT (**b**).

Another important point is the investigation of the effect of coating the MWCNTs with TiC nanoparticles on the in situ formation of Al_4_C_3_ upon thermal deformation of the composite powder under conditions of solid-phase consolidation. For example, in Reference [43], it was shown that Al_4_C_3_ can be formed in situ both in the form of an interface at the “MWCNT–aluminum matrix” interphase area and in the form of separately lying particles. Figure 8 shows AFM images of the surface of the bulk A_0.05CNT and A_0.05TiC/CNT composites. The insets show 2D Raman maps and spectra corresponding to the points given in the 2D maps. Thus, on spectra S1 and S3 (see Figure 8a), the Al_4_C_3_ lines are clearly observed. In this case, the intensity of the D- and G-lines was extremely low. On the contrary, D- and G-lines of high intensity are visible in spectrum S2 (see Figure 8a). This suggests that MWCNTs were present in this area. From the presented data, it follows that for A_0.05CNT composites, the in situ formation of Al_4_C_3_ near MWCNTs is possible.

At the same time, Raman spectroscopy of bulk A_0.05TiC/CNT composites showed the presence of D- and G-lines, as well as TiC lines (see Figure 8b). In this case, Al_4_C_3_ lines were not observed, i.e., TiC nanoparticles on the surface of the MWCNT hybrid reinforcement reduced the contact area of the MWCNTs with the matrix material (acting as a barrier interface), which also locally inhibited the reaction between the matrix and the MWCNTs.

**Figure 9:**
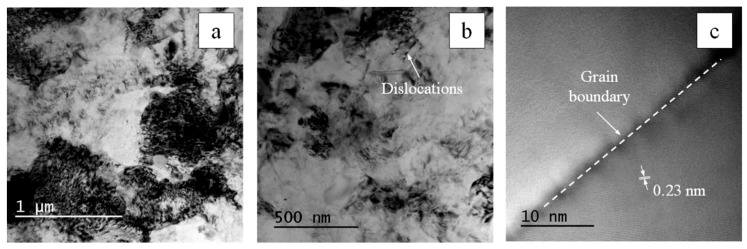
TEM image of the foil (**a**,**b**) prepared from the initial sample. The contrast in the micrograph corresponded to the contours of grain boundaries and other features (deformations, defects, and changes in thickness) of the sample. The characteristic grain size of the material was from hundreds of nanometers to a few microns. High-resolution image of aluminum grain boundary (**c**). The contrast of planes (111) of the aluminum crystal lattice is visible.

The TEM studies of bulk composites showed that, regardless of the type of reinforcement, the characteristic grain size of aluminum in the studied foil ranged from several hundred nanometers to a few microns. For example, Figure 9a,b shows typical TEM images of the fine structure of the A_0.05TiC/CNT aluminum matrix nanocomposite. A typical HRTEM image of the aluminum grain boundary is shown in Figure 9c. Judging by the contrast, there were no impurities at the boundary in the HRTEM image. Crystalline Al_2_O_3_ particles were also not detected. Nevertheless, taking into account that the oxygen content in the bulk composites A_0.05CNT and A_0.05TiC/CNT was 2–3 times higher than in A_un (see Table 2), we can assume the presence of a native oxide layer at the boundary of the powder particles [44,45]. However, the presence of such an oxide layer cannot make a significant contribution to strengthening, which was confirmed by the data in References [37,44]. It is difficult to judge the mutual orientation of the grains from the HRTEM images of the aluminum grain boundaries; however, in the diffraction patterns obtained from sufficiently large areas of the sample (more than several hundred nanometers in size), the diffraction pattern was close to that of a single crystal, although there was a slight mismatch between the reverse lattice grids of large grains and their small rotations when moving the sample. Therefore, summarizing the TEM data and the results of diffraction studies, we believe that the mutual misorientation of the grains was within the range of a few degrees.

AFM images of the surfaces of bulk composites A_0.05CNT and A_0.05TiC/CNT showed the presence of pores (see Figure 8). Moreover, the amounts and sizes of pores in the A_0.05TiC/CNT composites were higher than in A_0.05CNT, which also correlated with the results of measurements of the relative density of the samples. Measurements of the relative density of samples A_un, A_0.05CNT, and A_0.05TiC/CNT (by hydrostatic weighing) (see Table 2) showed that all bulk materials had a residual porosity of not more than 3%. The relative density of the samples could be improved by selecting methods (or combinations of methods) and conditions of consolidation.

Table 2 also shows the microhardness and modulus of the elasticity of powder and compact materials. The hardness of the composite powders was ~2 times higher than the hardness of bulk composites. This was consistent with the XRD data on a decrease in the half-width of diffraction peaks after isothermal exposure and can be explained by an increase in crystallite size and its effect on microhardness in accordance with the Hall–Petch relationship. It should also be noted that there was an increase in the elastic modulus of bulk composites compared to powder ones. This may also have been due to an increase in grain size during hot consolidation. This effect has been noted in studies of the effects of annealing temperature on the growth of copper grain size after intense plastic deformation [46,47]. In general, the elastic modulus of bulk composites A_0.05CNT and A_0.05TiC/CNT was comparable to the values obtained for AlMg5 reinforced with 0.5 vol % CNTs (90 GPa) [31] and Al reinforced with 0.5 wt % TiC/CNTs (90 GPa) [25] and exceeded the elastic modulus of materials obtained by consolidation of the initial matrix granules in the same conditions. The results obtained in this work (see Table 2) indicate that the use of additives of reinforcing carbon nanotubes in microconcentrations is no less effective for increasing the elastic modulus and therefore the stiffness of composite materials. Moreover, it is justified from an economic point of view. Moreover, small additives gave an elastic modulus comparable to or greater than the published values from a number of previous works.

**Figure 10:**
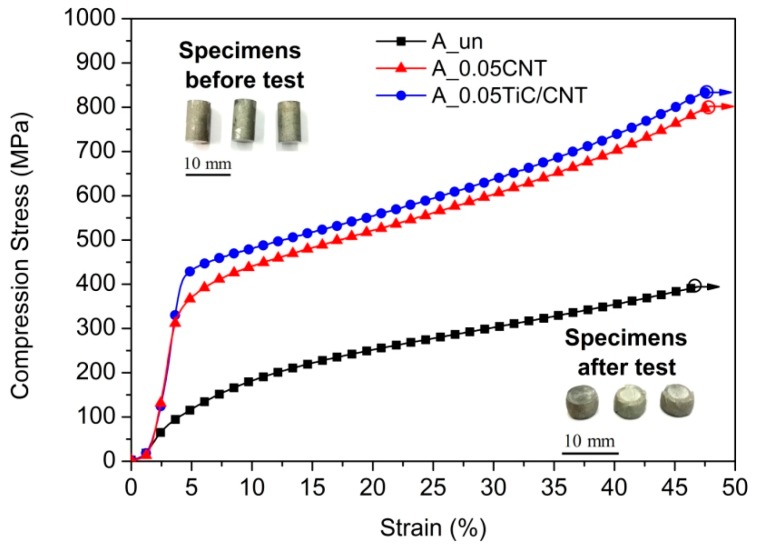
Typical compression stress–strain curves of different bulk AA5049-based materials. The insets show photographs of bulk specimens for compression tests before and after tests.

Typical stress–strain curves that were obtained during compression tests of bulk samples are shown in Figure 10. A comparative analysis of the obtained data showed that the conditional yield strength (YS) during compression increased from 80 MPa (A_un) to 420 MPa (A_0.05TiC/CNT). Sample A_0.05CNT had a YS value of 330 MPa that is lower than A_0.05TiC/CNT. It is also worth noting that an increase in strength did not lead to a decrease in the ductility of composite samples (at least during the compression test) to a degree of deformation of 50%, as can be seen from the extended curve profiles in Figure 10 (without breaks).

Thus, as a result of a fairly simple one-step process for producing Al/MWCNT composites (without hot isostatic pressing, extrusion, rolling, or the combined use of methods), a high density of consolidated samples was achieved (residual porosity not more than 3%). Taking into account the identical conditions of the synthesis processes and the subsequent consolidation of powders, the obtained data on the mechanical properties show the advantages of using reinforcement in the form of MWCNTs coated with TiC nanoparticles in comparison to MWCNTs. The results obtained for the strength characteristics were high for microcrystalline (fine and ultrafine grains) materials, while sufficient ductility of the metal base was preserved. Further searching for effective coating agents for multiwalled carbon nanotubes could be a promising direction for further improvement of the construction properties of new composite materials.

## 4. Conclusions

Using high-energy ball milling and uniaxial hot pressing, two types of bulk nanocomposites based on aluminum alloy AA5049 that were reinforced with microadditions of multiwalled carbon nanotubes and multiwalled carbon nanotubes coated with TiC nanoparticles were successfully produced. An analysis of the evolution of the structure of reinforcement both in the initial state and after ball milling and consolidation showed that the presence of TiC nanoparticles on the surfaces of MWCNTs on the one hand reduced the damage of reinforcement under the intense exposure of milling bodies. For example, the *I_D_/I_G_* ratio for powder composites synthesized under the same conditions was 0.90 and 0.57 when using the initial MWCNTs and MWCNTs coated with TiC. On the other hand, the obtained data showed that TiC nanoparticles located on the surfaces of MWCNTs acted as a barrier interface, locally inhibiting the reaction between MWCNTs and the matrix alloy and preventing the in situ formation of Al_4_C_3_ during solid-phase consolidation. A study of the mechanical properties of the synthesized nanocomposites showed that the use of reinforcement in the form of MWCNTs coated with TiC nanoparticles provided an increase in the yield strength by 21% compared to the initial MWCNTs. Moreover, the conditional yield strength of the nanocomposites, even with microadditives of 0.05 wt % of reinforcement, was 4–5 times higher (depending on the type of reinforcement) than that of the matrix alloy AA5049. In addition, an increase in the elastic modulus was noted, indicating an increase in the stiffness of nanocomposite materials compared to the matrix alloy by 17% and 25% with additions of MWCNTs and TiC/MWCNTs, respectively.

## Figures and Tables

**Table 1:** Investigated compositions. CNTs: carbon nanotubes; MWCNTs: multiwalled carbon nanotubes.

Ref. Name	Reinforcement	Process Control Agent
A_un (unmilled)	–	–
A_0.05CNT	0.05 wt % MWCNTs	0.2 wt %
A_0.05TiC/CNT	0.05 wt % TiC/MWCNTs	0.2 wt %

**Table 2:** The properties of composites in powder and bulk states.

Ref. Name	Powder		Bulk			
	Microhardness (GPa)	Elastic Modulus (GPa)	Relative Density (%)	Microhardness (GPa)	Elastic Modulus (GPa)	Oxygen Content (wt %)
A_un (unmilled)	–	–	98.19	0.68	72.3	0.05
A_0.05CNT	2.08	75.6	98.83	1.03	86.8	0.15
A_0.05TiC/CNT	2.06	71.1	97.34	1.09	96.7	0.12
